# Spectrum Sensing, Clustering Algorithms, and Energy-Harvesting Technology for Cognitive-Radio-Based Internet-of-Things Networks

**DOI:** 10.3390/s23187792

**Published:** 2023-09-11

**Authors:** Xavier Fernando, George Lăzăroiu

**Affiliations:** 1 Intelligent Communication and Computing Laboratory, Toronto Metropolitan University, Toronto, ON M5B 2K3, Canada; george.lazaroiu@spiruharet.ro; 2 Department of Economic Sciences, Spiru Haret University, 030045 Bucharest, Romania

**Keywords:** cognitive radio, internet-of-things networks, spectrum sensing, clustering, energy harvesting

## Abstract

The aim of this systematic review was to identify the correlations between spectrum sensing, clustering algorithms, and energy-harvesting technology for cognitive-radio-based internet of things (IoT) networks in terms of deep-learning-based, nonorthogonal, multiple-access techniques. The search results and screening procedures were configured with the use of a web-based Shiny app in the Preferred Reporting Items for Systematic Reviews and Meta-analysis (PRISMA) flow design. AMSTAR, DistillerSR, Eppi-Reviewer, PICO Portal, Rayyan, and ROBIS were the review software systems harnessed for screening and quality assessment, while bibliometric mapping (dimensions) and layout algorithms (VOSviewer) configured data visualization and analysis. Cognitive radio is pivotal in the utilization of an adequate radio spectrum source, with spectrum sensing optimizing cognitive radio network operations, opportunistic spectrum access and sensing able to boost the efficiency of cognitive radio networks, and cooperative spectrum sharing together with simultaneous wireless information and power transfer able increase spectrum and energy efficiency in 6G wireless communication networks and across IoT devices for efficient data exchange.

## 1. Introduction

Cognitive radio has been developed due to spectrum scarcity and diminished exploitation [1] of allocated spectral resources by registered users, and should have more extensive spectral awareness that can be attained by taking advantage of more spectral options available for selection over a wideband spectrum. Cognitive radio technology can enhance spectrum use and mitigate spectrum scarcity across wireless networks [2]: spectrum sensing assists secondary users in identifying spectrum holes and accessing the unoccupied spectrum. Intelligent cognitive approaches can improve 5G network spectrum deployment to find a solution to spectrum congestion and thoroughly optimize radio efficiency. In cognitive radio networks, opportunistic spectrum access is typically harnessed for secondary users to identify primary user spectrum usage and detect spectrum holes for transiently sharing spectrum resources in data distribution across unoccupied channels. Access to an ample series of spectrum resources constitutes a main growth determinant for leveraging large-scale internet of Things (IoT) networks and first-rate mobile broadband services, while the spectrum may be a hindering element in 5G communication expansion.

Cognitive radio technology can dynamically distribute the unlicensed spectrum [3] for IoT-connected devices. Diverse wireless devices can access the primary user licensed spectrum. Cognitive-radio-based IoT networks assist interconnected devices [4] in efficiently leveraging spectrum resources. Cognitive radio technology can facilitate streamlined and opportunistic spectrum band utilization by use of vacant licensed channels [5], articulating a massive spectrum that can further coherent extensive implementation for IoT networks. Spectrum sharing across cognitive radio networks develops dynamic spectrum access, where cognitive radio users can opportunistically use any area of the spectrum, resulting in coherent IoT deployment and enabling massive IoT device interactions by a media access control procedure that harnesses the available spectrum resources across cognitive radio-IoT networks.

IoT-based large-scale wireless connections can bring about a serious spectrum scarcity issue [6], and thus, cognitive IoT, by integrating short-packet transmissions, can solve the issue efficiently. The research problem of this uniquely designed systematic review was whether there are robust enough correlations between spectrum sensing, clustering, and energy harvesting so as to optimize cognitive-radio-based IoT networks in terms of deep-learning-based nonorthogonal multiple-access techniques. The manuscript was organized as follows: methodology (Section 2), spectrum sensing for cognitive-radio-based IoT networks (Section 3), clustering algorithms for cognitive-radio-based IoT networks (Section 4), energy-harvesting technology for cognitive-radio-based IoT networks (Section 5), discussion (Section 6), conclusions (Section 7), specific contributions to the literature (Section 8), limitations and further directions of research (Section 9), and practical implications (Section 10).

## 2. Methodology

**Figure 1:**
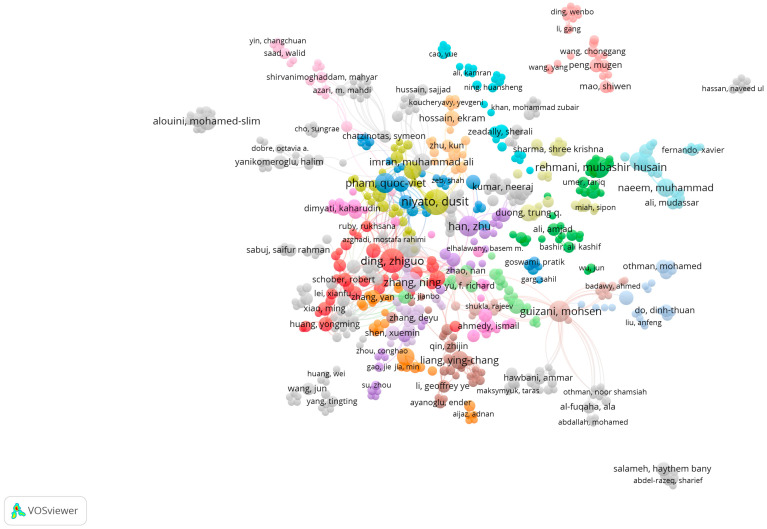
VOSviewer mapping of spectrum sensing, clustering algorithms, and energy-harvesting technology for cognitive-radio-based IoT networks regarding co-authorship.

**Figure 2:**
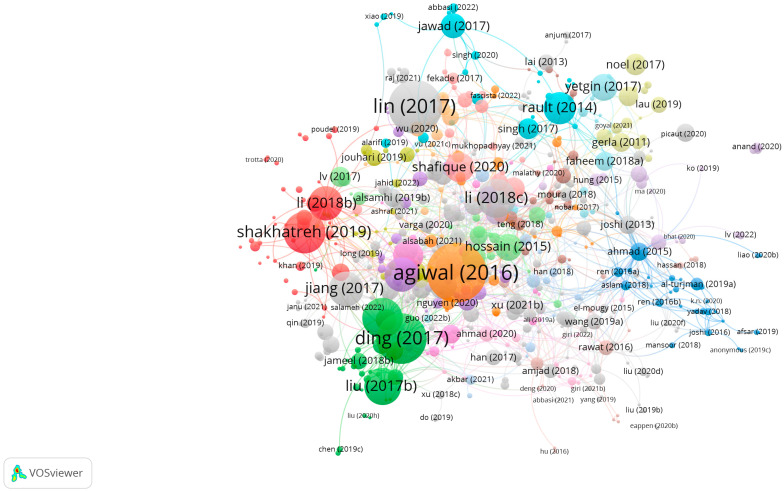
VOSviewer mapping of spectrum sensing, clustering algorithms, and energy-harvesting technology for cognitive-radio-based IoT networks regarding citation.

**Figure 3:**
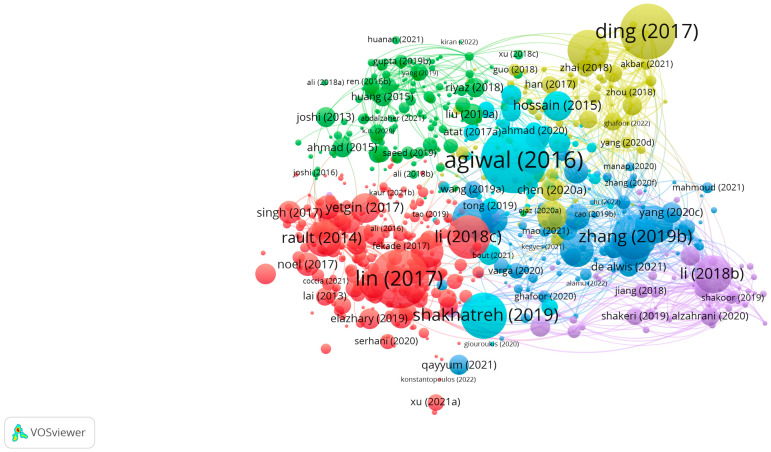
VOSviewer mapping of spectrum sensing, clustering algorithms, and energy-harvesting technology for cognitive-radio-based IoT networks regarding bibliographic coupling.

**Figure 4:**
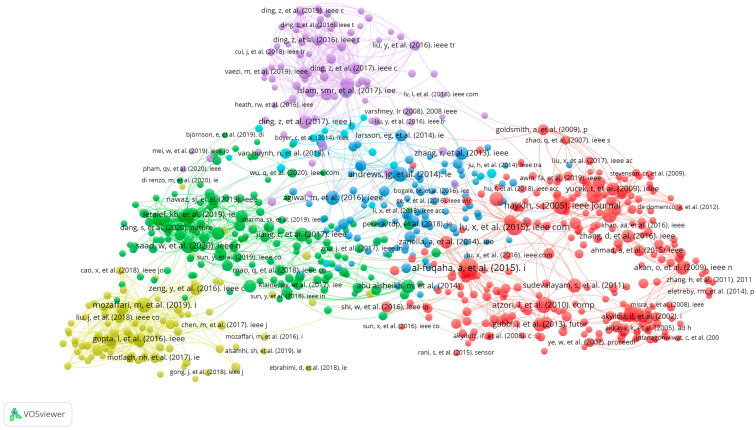
VOSviewer mapping of spectrum sensing, clustering algorithms, and energy-harvesting technology for cognitive-radio-based IoT networks regarding co-citation.

**Figure 5:**
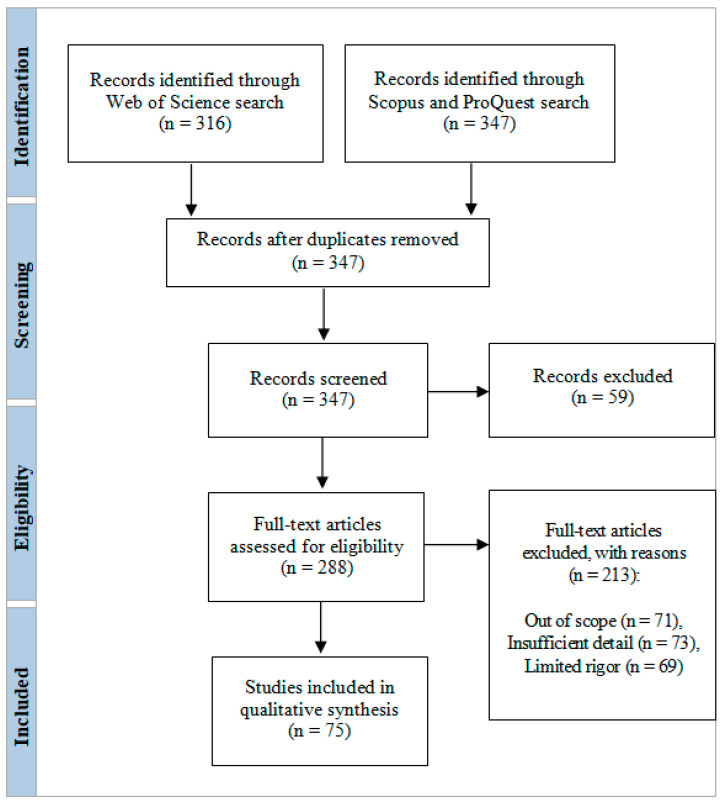
PRISMA flow diagram describing the search results and screening.

The search results and screening procedures were configured by use of a web-based Shiny app in the Preferred Reporting Items for Systematic Reviews and Meta-analysis (PRISMA) flow design. In July 2023, a quantitative literature review of major scholarly databases (ProQuest, Scopus, and the Web of Science) was carried out, and the search terms were: “cognitive radio-based IoT networks” + “spectrum sensing”, “clustering algorithms”, and “energy harvesting technology”, with the analyzed research being published between 2019 and 2023; only 347 sources qualified for initial inclusion (Table 1). A total of 75 final, mainly empirical, papers were selected for analysis. AMSTAR, DistillerSR, Eppi-Reviewer, PICO Portal, Rayyan, and ROBIS were the review software systems harnessed for screening and quality assessment, while bibliometric mapping (dimensions) and layout algorithms (VOSviewer) configured data visualization and analysis (Figure 1, Figure 2, Figure 3, Figure 4 and Figure 5).

As increasing demands for significant data rates overload standard radio frequency technologies [7], millimeter waves and cognitive radios can surmount the spectrum scarcity and capacity constraints of the standard radio frequency systems. Flexible and effective smart cognitive networks can adjust to the surrounding environment and use the available resources exemplarily, optimizing user performance and preserving streamlined resource utilization. A low-battery reconfigurable multimode low-noise amplifier can be designed for heterogeneous narrowband and wideband operations [8] while enabling coextensive input matching and output load arrangement. Reconfigurable intelligent surfaces assist network operators in monitoring radio waves to remove the detrimental consequences of natural wireless propagation [9], and through the use of nonorthogonal multiple access, adequate transmissions can be provided. Radio resources’ limited availability together with interference elevated levels reduces dense network spectrum reuse [10], but cognitive radio technology can improve spectral efficiency through enabling low-priority unlicensed users to have the same spectrum as high-priority licensed users (the transmission parameters are adjusted according to the application requests, resulting in streamlined spectrum management and interference reduction across 5G cellular networks). Cognitive-radio-based spectrum sharing approaches can enable 5G cellular network services. In addition, 5G cognitive-radio-enabled spectrum access technology can optimize the spectrum and energy efficiency, together with the secrecy of networks, by progressively adjusting its transmission parameters. An actor–critic reinforcement learning strategy can enhance the cognitive network prolonged throughput [11]: by interacting and assimilating knowledge from the environment across various time slots, the cognitive base station can exemplarily assign the volume of transmission energy for each secondary user in conformity with the residual energy and primary channel availability.

Cognitive radio networks can articulate multipurpose spectrum management [12] by enabling secondary users to provisionally use the licensed spectrum lacking a primary user. Each secondary user carries out spectrum sensing and transmits the collected data to the centralized controller in a backward induction approach. Complex and heterogeneous industrial IoT devices and networks [13] have various quality-of-service demands (e.g., ultra-reliable low-latency communications and elevated sharing data rates), while spectrum and energy resources are in low volumes. A heterogeneous radio frequency can provide wide-area coverage. Ambient backscatter communication enables secondary transmitters to share information [14] by modulating and displaying ambient radio frequency signals. IoT sensor devices in relation to data transferring and sorting [15] can be harnessed as a wireless sensor network across a mesh topology. Sensor network capabilities can be optimized by amplifying power use, bandwidth, and soundness in the mesh topology. IoT data place a massive load on the network components attempting to share input among end users. Data mining algorithms, together with multiagent and self-organizing technologies, shape sensory systems. Data fusion can curtail the volume of data sharing and energy use across wireless sensor networks [16], but data fusion schemes typically bring about further delay overhead and power use. In order to enhance wireless sensor network performance, a hybrid delay-aware clustering-based intelligent data fusion algorithm merges single-layer and multilayer cluster structure upsides, to harmonize the tradeoff between intermission and energy use in wireless sensor networks and the performance oscillation under heterogeneous fusion rates.

Cognitive radio can optimize wireless communication spectral efficiency [17] by enabling spectrum sharing among primary and secondary users. Intelligent reflecting surfaces/reconfigurable intelligent surfaces can improve wireless communication spectral efficiency through channel environment reconfiguration. Cognitive radio networks and multiple-access schemes assist in high-speed connectivity and in optimal dynamic spectrum distribution across 5G networks [18] as regards spectrum and energy efficiency, scalability, and delay. Users can share data streams at the same time under maximum capacity limitations through multiple-access schemes, while inactive spectrum holes can be leveraged in an opportunistic way through the use of cognitive and software-defined radios. While nonorthogonal multiple and space division multiple access are instrumental in multiplexing, handling the counterproductive spectrum use generated by orthogonal multiple-access schemes, rate splitting multiple access can improve spectrum efficiency considerably. Through cutting-edge spectrum management approaches, increased spectral performance can be attained while fulfilling the large-scale connectivity requirement. The quality of service demands of nonorthogonal multiple-access-enabled multifarious networks can enable power distribution and user scheduling schemes. Spectrum sensing techniques should be deployed in nonorthogonal multiple-access-enabled cognitive radio networks to identify exploitable frequency bands. When nonorthogonal multiple-access techniques are not used across a primary network, the established spectrum sensing algorithm can be leveraged, as under other conditions, nonorthogonal multiple access supplies services for heterogeneous primary users. Space division multiple access harnesses a linear precoding scheme to differentiate spatial domain users and involves considering surplus multiuser fading as noise. With nonorthogonal multiple access, users should decode interference while receiving the information, which furthers the computational intricacy of correlative signal processing. Cognitive ambient backscatter communication can assist the green IoT as regards serious energy and spectrum limitations [19]: a backscatter device can articulate communications from the concomitant spectrum and radio-frequency source sharing.

The traffic behavior modeling of impermanent spectrum use for IoT applications across collective bands [20] can shape current interference by leveraging a software-defined radio to continuously inspect the transitory episodes of IoT transmissions, and collecting the time-series data transferred to power spectral density so as to take out the identified occupancy. An unsupervised machine learning technique can improve standard energy detection strategies. Lean data sensing together with wireless power transfer can articulate sustainable and sound performance [21] across industrial IoT networks. The thoroughness of on-demand data gathering and wireless power transfer can be heightened by streamlined time management of IoT nodes and customized energy transmitters. An energy-aware mode switching approach can assist IoT nodes in carrying out either lean data sensing or customized wireless power transfer. An IoT node time management scheme can increase the effectiveness of the IoT nodes by including unused energy and energy demanded for sensing operation and taking into account sensing task reliability. An energy transmitter time management scheme for IoT nodes can reduce charging expenses while keeping IoT nodes adequately charged. Deep-learning-method-based passive signal detection [22] can optimize cognitive-radio-based detection across low-signal-to-noise environments. Convolution neural networks and long short-term memory algorithms can be leveraged in signal frequency and time domain feature extraction.

## 3. Spectrum Sensing for Cognitive-Radio-Based IoT Networks

Cognitive radio networks reach a compromise [23] between energy and spectrum sensing efficiency. Spectrum sensing is pivotal in cognitive radio technology [24] whose sensing performance is typically assessed as false-alarm and detection probabilities. Spectrum sensing can optimize spectrum use [25] across cognitive radio networks. Spectrum-prediction-based sensing schemes reduce the energy use of the sensing module across cognitive radio networks [26] by inferring the status of spectrum before carrying out effective physical sensing. The joint mode of spectrum prediction can surmount local prediction model issues. Spectrum sensing, the energy-consuming procedure that should be decreased because of resource limitations [27], enables cognitive users to distinctively detect unexploited radio spectrum segments and keep interference to primary users from happening. Cognitive-radio-enabled IoT cellular networks, incorporating heterogeneous primary user base stations and secondary user devices as IoT smart objects [28], carry out collective spectrum sensing and the appropriate spectrum distribution to the soliciting secondary user-IoT devices by use of an intelligent fusion center. Short-time Fourier transform and convolutional neural network algorithms can assist spectrum sensing in finding a solution to the spectrum resource scarcity [29] through signal sample time–frequency domain information.

A cognitive radio network comprises primary and secondary users [30]: the latter sense the spectrum band to swiftly use the white space, resulting in spectrum efficiency improvement. Long short-term memory networks are satisfactorily applicable for time-series data. Reliable spectrum sensing assists cognitive radio networks [31] in identifying and deploying unused and underused frequency bands. By employing historical detection data, online learning algorithms integrating the optimum decision threshold clarify the occurrence or nonappearance of the primary user [32], boosting the spectrum sensing performance and reducing the total error probability. Cognitive radio networks detect band vacant spots [33] by adequately sensing and distributing the spectrum to the demanding users. Multiobjective brainstorm optimization algorithms can manage the energy–throughput trade-off in cognitive radio networks and reduce the packet error rate [34], as throughput maximization can lead to high energy consumption. The spectrum sensing performance is improved with the increased probability of detection. Spectrum sensing and insufficient battery capacity can minimize system performance across cognitive radio networks [35], and thus, wireless-powered communication requires energy efficiency optimization.

Cognitive-radio-based IoT systems [36] develop on coherent spectrum sensing and sharing. Software-configurable radio having dynamic spectrum assistance constitutes the intrinsic feature of cognitive radio [37] whose coaction with wireless sensor networks makes it possible for the sensor nodes to use and share application data throughout licensed primary user free channels. Improved operations can be attained with opportunistic spectrum access by reducing the channel access incompatibilities and control message overhead postponement. The cognitive radio spectrum sensing performance [38] necessitates detection accuracy as regards whether primary users are active or not. Secondary user teamwork can optimize spectrum detection operations throughout cognitive radio networks. As incessant spectrum sensing significantly decreases the duration of a network encompassing energy-restricted cognitive radio nodes [39], precise approaches as regards predicting spectrum occupancy optimize energy efficiency. Intelligent reflecting surface-optimized energy detection [40] is pivotal in spectrum sensing performance across cognitive radio networks.

Spectrum sensing is decisive [41] in cognitive radio system operations. Matched filtering is typically harnessed for signal detection across a particular band of spectrum for an identifiable primary user signal. Spectrum sensing aims to increase the detection operations [42] of secondary users across cognitive radio networks. All secondary users provide sensing assessment to the fusion center for the eventual decision in relation to the operations of primary users in cooperative spectrum sensing. The teamwork among massive volumes of secondary users can generate overhead for the fusion center. Cooperative spectrum sensing schemes can find a solution to the hidden terminal issue and reduce multipath fading and shadowing effects [43], optimizing the sensing performance and throughput across cognitive radio networks. Increasing the volume of cooperative secondary users results in intensified communication overhead and thus in energy consumption elevation of cognitive radio networks. Cognitive radio and multiple-access techniques can enhance spectral efficiency and enable massive connectivity [44]: spectrum sensing accuracy determines spectrum utilization efficiently through multiple-user cooperative spectrum sensing.

Deep-learning-based cognitive radio technology can be harnessed throughout wireless communication systems [45], increasing energy efficiency for shared spectrum sensing by incorporating reinforcement learning algorithms and graph neural networks. Energy detection is decisive in terms of time and resource efficiency [46], but its performance is unsatisfactory in low-signal-to-noise ratio channel circumstances, due to its marginal hardware complexity and the nonexistence of inferable licensed user information. Cooperative sensing can mitigate the energy detection sensing performance issue in IoT networks, but relevant detection cannot be attained in detrimental channel environments by deploying incompatible IoT applications. Stochastic resonance can elevate spectrum sensing performance in weak signal detection in cognitive radios. Artificial-intelligence-enabled intelligent radio [47] can be optimized to smoothly leverage the insufficient spectrum resources and to exemplarily connect and configure large-scale wireless devices in spectrum sensing and sharing-based communication systems. Deep- and machine-learning-based automatic modulation recognition [48] can carry out spectrum sensing and efficiency across cognitive radio networks and can articulate a lean network resource management.

## 4. Clustering Algorithms for Cognitive-Radio-Based IoT Networks

The design and advancement of energy- and spectrum-efficient proposals, such as cognitive radio sensor networks [49], articulate IoT, with clustering optimizing the energy consumption. IoT enabling sensor-based network device connectivity is subjected to critical data exchange interference [50] due to unlicensed spectrum overcrowding. Cognitive radio IoT networks can solve the spectrum scarcity issue, but the sensor nodes use considerable energy throughout dynamic spectrum sensing and switching. Channel spectrum sensing can optimize energy efficiency across clustered cognitive radio IoT networks. A cognitive radio sensor network senses event signals and conjointly interconnects in a multihop mode [51] across variably operational spectrum bands. Nodes playing a part in cognitive radio sensor networks grasp the network environment and have autonomous decision making in relation to throughput intensification, discontinuity, and energy reduction, while clustering algorithms extend the network lifetime. 

Clustering and data aggregation are decisive in IoT-based wireless communication [52], while energy efficiency can be attained by cognitive networks. Sensor node insufficient energy and data sharing channel-related operations [53] affect energy performance across cognitive radio sensor networks. Unequal clustering can level the energy use among the clusterheads to extend the network lifetime. Energy- and spectrum-aware unequal clustering surmounts energy and spectrum for prolonging cognitive radio sensor network lifetime, while enhancing equity by establishing residual energy stability among the sensor nodes and optimizing the network lifetime by decreasing the energy use. The spectrum holes can be predicted through the use of deep belief network algorithms. A shared sensing network comprises heterogeneous nodes intercommunicating [54] in relation to the specific spectrum sensing output. The secondary user nodes of each cluster identify the spectrum, leading to incessant power consumption in cognitive radio sensor networks. 

Spectrum dynamics and energy use can be assimilated in network-stability-aware clustering [55] that coherently handles interactions across cognitive radio sensor networks. Cognitive radio chiefly addresses the streamlined harnessing [56] of available spectrum bands. Cognitive radio networks should integrate spectrum management approaches to allocate the unutilized spectrum band to the cognitive radio users by conforming to a series of sensing-related operations. A cooperative spectrum sensing strategy with a feature-based cluster classifier can reduce the time to accomplish optimal cognitive radio communications. Such a classifier assimilates states and transitions across radio frequency settings, in addition to primary user operations at constant periods to assist the spectrum decision approach. A hybrid strategy integrating clustering and expected maximization and reinforcement learning algorithms improves system operations with precise sensing outcomes, and by detecting the optimum spectrum band by use of the hierarchical access model deploying the interweaving technique, energy use is reduced. 

Clustering arranges nodes into groups [57] so as to improve cognitive radio sensor network connectivity and soundness. Contingent upon the channel availability, spectrum-aware clustering algorithms cannot generally attain optimal clustering. Considering diverse relevant factors, to set up the optimal clustering constitutes a difficult task in network operation enhancement. Weighted clustering metric-based spectrum-aware clustering algorithms can lead to optimal clustering, concomitantly assessing temporal–spatial correspondence and the confidence level, and unused energy is deployed to decide on clusterheads and ally member nodes. The clusterhead sensing spectrum significantly diminishes spectrum sensing energy use and increases data sharing opportunity after clustering. A cluster-based cognitive industrial IoT can enhance spectrum use by sensing and accessing the inactive spectrum [58]: the clusterheads carry out cooperative spectrum sensing to obtain convenient spectrum, while the nodes use the nonorthogonal multiple access. Transmission performance can be optimized by clustering algorithms, while energy balance is determined by clusterhead alternation. The nonorthogonal multiple access configured for the cluster-based cognitive industrial IoT can efficiently enable the transmission operation of each node. 

A coherent and green machine-learning-based dynamic clustering mechanism integrating power demand and data volume can assist cognitive IoT networks in terms of intelligent processing, secure delivery, and far-reaching awareness [59], leading to energy-efficiency-based real-time implementations and information loss avoidance. Machine learning techniques and clustering algorithms improve cognitive radio network performance [60], solving the radio spectrum underutilization issue efficiently through the use of learning and reasoning capabilities. Bayesian-learning-based intelligent clustering cooperative spectrum sensing can optimize the performance of cognitive radio networks lacking a primary user, in serious fading and shadowing circumstances of the sensing channel [61], while also minimizing the rate loss and shared overhead. Cognitive radio technology and the reaction–diffusion biological mechanism can configure streamlined cognitive IoT spectrum allocation and adequate bioinspired algorithm-based clustering performance [62], enhancing clustered throughput and decreasing convergence time, communication delay, and computation complexity through intelligent service provisioning, reliable wireless communication, and automatic network operation.

Cognitive wireless sensor networks can harness the inactive authorized frequency band to find a solution to the spectrum resource scarcity issue [63]: by leveraging the spectrum hole, spectrum sensing technology can deteriorate the synchronic interference and improve the entire sensor network performance. As a result of the insufficient battery energy and low sensor node processing capacity features, the energy efficiency and the spectrum sensing performance have to be optimized. Particle swarm optimization algorithms can assist cognitive wireless sensor networks by integrating a cooperative spectrum sensing approach in relation to false alarm and detection probability, enhancing the system throughput and energy efficiency. Cognitive radio and radar systems leverage dynamic spectrum access techniques to solve spectrum congestion issues due to increased data traffic [64]: dynamic spectrum access approaches share the radar and communication system spectrum. Machine-learning-based efficient resource allocation can improve dynamic clustered IoT network power management and machine-to-machine communication [65] in terms of spectrum management.

Cognitive-radio-network-based real-time high-speed communication systems [66] require effective resource distribution, spectrum sensing, ubiquitous computing services, and power use issues. Backtracking search algorithms and cooperative node selection can decrease computation complexity and energy consumption. Genetic algorithms and dynamic clustering techniques [67] are pivotal in conserving energy throughout IoT network planning and designing procedures. High-energy clusterheads enable optimal data sharing in wireless sensor networks. Cognitive radio technology develops user communication reliability and the medium by coherent dynamic spectrum exploitation [68] in terms of spectrum distribution and channel access, optimizing radio resource use rate. The internet of spectrum devices, through spectrum data analytics and accurate collective time–frequency spectrum predictions, articulates spectrum-monitoring and spectrum-utilizing device networks [69] to facilitate a coherent spectrum distribution and management pattern for 5G wireless networks, improving the inference performance.

Metaheuristic algorithms and deep-neural-network-based clustering techniques [70] can improve IoT-related data clustering reliability and computation times. Intelligent edge computing and deep learning convolutional neural networks [71] can assist resource-constrained IoT devices, enhancing communication volume and inference latency through data analytics. Deep-neural-network-based clustering techniques can maximize wireless sensor network functioning period in IoT applications [72]: by modifying individual sensor node roles, energy consumption is reduced and the network lifetime is extended (relevantly, computation and message overheads also decrease). A hybrid delay-aware clustering-based intelligent data fusion algorithm [16] can optimize wireless sensor network performance by integrating the single-layer and multilayer cluster structure upsides. The energy-efficient clustering and the dynamic clusterhead reselection algorithms can cut down the network delay, energy use, and load balancing while increasing the network lifetime.

## 5. Energy-Harvesting Technology for Cognitive-Radio-Based IoT Networks

Energy harvesting and cognitive radio technologies can assist wireless sensor networks [73], extending the operational activity of the sensor node and mitigating the unlicensed spectrum congestion issue. Carefully distributing and organizing limited network resources are decisive because of energy-harvesting process unpredictability and primary user behavior randomness. Cognitive radio and energy-harvesting strategies [74] are instrumental in spectrum reutilization and lifetime extension for standard wireless networks. Energy-harvesting cognitive radio networks comprising multiple primary and secondary users integrate energy and joint cooperation modes. Sensing energy and data manageability [75] shape the secondary performance of energy-harvesting cognitive radio networks.

As IoT sensor and devices use a massive volume of power in data transmission [76], radio frequency energy harvesting can assist self-sustainable wireless systems whose system rate loss is caused by external interference factors. The cognitive industrial IoT can increase convenient spectrum resources [77] by harnessing the spectrum authorized to primary users with the aim of not discontinuing primary user communications, but increased spectrum sensing and prolonged operations may use much energy. Wireless energy harvesting can acquire the radio frequency energy of a primary user signal, and energy-efficient resource distribution in heterogeneous spectrum access modes can optimize the standard transmission rate of the cognitive industrial IoT and meet energy-saving demands. Cognitive radio techniques can be harnessed for wireless power transfer, power consumption reduction, and energy harvesting [78] throughout the sensing, interaction, and computation elements of IoT nodes. Backscatter communication can facilitate green IoT operations through collective wireless communication and sensing.

Availability and ultrareliability demands, together with energy-harvesting technology and dynamic spectrum access, impose specific performance compromises [79], typifying sustainable and self-sufficient IoT networks, integrating sensing time, energy availability, transmission diversity, volume of data frame packets, and spectrum accessibility. Energy and spectrum resource scarcity, energy harvesting and cognitive radio technologies, and wireless devices and system expansion [80] shape deep-learning-based IoT network performance. Energy harvesting and cognitive radio technologies design deep-learning-based IoT networks [81]: spectrally and energy-efficient transmission schemes should be articulated in large-scale connection and device support. Spectrum reutilization and lifetime extension assist energy-harvesting cognitive radio networks [82]: the energy provision of a primary transmitter can be reduced while meeting the requirements of minimal-throughput networks and users.

A deep-Q-learning based algorithm can be deployed across energy-harvested cognitive radio networks with the aim of optimal resource distribution [83]: primary users’ network channel resources also allocated to secondary users and energy harvesting enable cognitive radio network nodes to acquire environment energy to achieve operation sustainability. The amount of environmental energy necessitates dynamic resource distribution to straighten out network and throughput capacity. A deep-Q-learning-based algorithm can enhance energy-harvested cognitive radio network resource distribution so it surpasses low quality of service, massive state–space systems, energy and interference limitations, and slow convergence. Nonorthogonal multiple access, energy-harvesting technology, and cognitive radio systems can [84] optimize the energy and spectral efficiency of the 5G network for IoT wireless sensor communication support. Deep-reinforcement-learning-based distributed multidimensional resource management algorithms can be decisive in intelligent frequency, the joint spectrum, and energy and time resource management, and thus decrease secondary sensing user data packet losses while meeting the limitations on the maximum buffer capacity, transmitting power, charging battery capacity, and primary and secondary sensing user minimum data rate.

The spectral and energy efficiency of device-to-device communication can be enhanced by employing cognitive radio systems and radio frequency energy-harvesting technologies [85] while stabilizing increased data rates and reducing power use in 5G communication networks. The primary and secondary transmitters interact with receivers across energy-harvesting amplify-and-forward relays for nonorthogonal multiple-access-based multicast cognitive radio networks [86], attempting to synchronously optimize the network sum-rate, decrease energy use, and fulfill quality-of-service limitations. A low-complexity solution approach can appropriately find a solution to the power distribution issue over each relay, and subsequently decide on the relay optimizing the network goal function, while adjusting spectrum and energy efficiencies and configuring the optimal network sum-rate and lower computational complexity. Cognitive-radio-based nonorthogonal multiple-access systems can satisfy IoT-driven 5G network requirements [87]: power domain nonorthogonal multiple access enables multiple users to share orthogonal resource blocks, while cognitive radio technology facilitates opportunistic bandwidth use, and thus, secondary users can access the licensed spectrum frequency while the operations performed by primary users are not interrupted.

The massive increase in smart devices articulating content-centric data traffic amplifies cellular-network-based group communication services [10]: multicasting can handle resources adequately and enable simultaneous common data sharing to a significant volume of users. The bandwidth large-scale communication demands of multimedia multicast applications necessitate exemplary spectral resource use, while the connectivity, immense capacity, and ultra-low-latency demands of the content-centric applications have led to closer content to the users and short-range communication deployment, resulting in cellular network compaction. Hybrid overlay–underlay cognitive radio networks can enable both secondary user constancy and satisfactory total throughput [88]: in cooperative cognitive radio IoT networks, secondary users can serve as a relay to assist in the primary transmission and can access the spectrum for data sharing in overlay mode. The secondary nonrelay nodes share data simultaneously with the active primary user in underlay mode. Cognitive radio networks can solve the spectrum scarcity issue in wireless communication systems [89], enabling dynamic opportunistic use by secondary users when primary users do not use the spectrum. Spectrum sensing facilitates the presence detection of primary users, while routing assists in coherent device communication, with both of them being pivotal in cognitive radio network IoT-based systems.

Nonorthogonal multiple access can enhance 5G cellular network throughput and spectrum efficiency and facilitate ultrareliable and low-latency communications [90], articulating spectrum- and energy-efficient transmission schemes across clustered IoT smart devices and massive system connectivity, while energy-harvesting algorithms and random access techniques can decrease signaling overhead, energy use, and packet latency. A game-based fair resource allocation algorithm can enable stable cooperation between primary users and secondary users [91] across wireless powered cooperative cognitive radio networks through streamlined resource allocation. Nonorthogonal multiple access can bring about spectrum efficiency [11] across wireless networks. In an uplink nonorthogonal multiple-access cognitive system, secondary users can collectively transfer data, throughout the same spectrum resources, to the cognitive base station, and uninterrupted interference discontinuation is applied to retrieve secondary-user-transmitted signals. A wireless energy harvester can extend secondary users’ operations. 

Energy-harvesting-powered cognitive machine-to-machine networks can mitigate the intensifying deficient spectrum, as a result of large-scale smart devices and simultaneous access demand that bring about operational deterioration and massive energy use [92], by ensuring the quality of service and leading to green communication through deep-reinforcement-learning-based algorithms in terms of energy efficiency optimization. The end-to-end throughput can be assessed and enhanced in wireless-powered cognitive IoT networks through the use of a well-organized deep-neural-network-based relay selection scheme [93]: multiple energy-harvesting relays are harnessed unselectively to enable data sharing to multiple users from a source node across energy-harvesting circuit practical nonlinearity, decreasing computational complexity significantly. Cognitive radio technology and nonorthogonal multiple-access techniques [94] can assist energy harvesting in spectral and energy efficiency optimization across IoT networks.

## 6. Discussion

Cognitive radio technology and nonorthogonal multiple-access techniques [95] can solve the spectrum scarcity issue and enable relevant enhancement in terms of spectral efficiency, particularly in combination. Nonorthogonal multiple access constitutes a feasible wireless access scheme [25] in 5G wireless communication systems. A distributed cognitive cellular network can incorporate machine learning and cognitive radio technology in a multiagent system [96] for efficient dynamic spectrum resource transfer. As heterogeneous radio access networks with distinct features may operate together, cognitive radio networks have to opt for the optimal network [97] through intelligent spectrum management and machine learning techniques. Green cognitive radios can generate high energy efficiency [27] in wireless communications.

Data-driven and wireless communication technologies, by integrating a large volume of end users, have led to a crowded radio spectrum [48], while groundbreaking electromagnetic environments have generated unstable and unsound services. The insufficient copresence capabilities of IoT wireless standards [98] bring about counterproductive spectrum use and collective performance deterioration. Industrial IoT applications have strict quality-of-service demands and low error tolerance. Dynamic spectrum access techniques can relevantly take advantage of interference mapping across various radio space dimensions. Low-power wide-area networks constitute the main communication platform harnessed in IoT applications [99]: spectral congestion issues can be straightened out by cognitive radio technologies. By exploiting spectrum holes [100], cognitive radio improves radio resource use. Pivotal in cognitive radio networks, dynamic spectrum allocation algorithms grant cognitive users access to convenient frequencies and bandwidths to interact opportunistically and to reduce primary and secondary user interference. Reinforcement learning techniques, swiftly inspecting the volume of data in a model-free way, significantly ensure dynamic spectrum allocation operations. 

Wireless sensor networks assist IoT devices [101] in physical condition observing and recording. The sensor nodes are self-governing and articulate an intercommunication topology in an improvised way, but have limited resources for energy administration, processing power, and data storage and sharing. Radio-frequency-based far-field wireless power transfer can optimize extensive IoT network power [102], but as radio frequency communication signals integrate both information and energy, simultaneous wireless information and power transfer can wirelessly charge IoT devices. Cognitive and software-defined radio enhance spectrum use and optimize the leverage of frequency bands between users [103], as there is an incessant request for wireless devices that can adjust to various channel features, while functioning on high distribution data-rates deploying heterogeneous communication bands. Deep-learning-based cognitive radio networks are pivotal in spectrum hole grasping and location [2], and thus, primary and secondary users can smoothly distribute network spectrum resources among them, enabling ubiquitous connectivity across the internet-of-things-driven communication infrastructure through 5G wireless technologies.

## 7. Conclusions

Multiple-access schemes that can increase the volume of users transmitting over a medium, together with cognitive radio networks that enable harnessing vacant frequency bands of a spectrum in a dynamic or opportunistic manner [104], can harmonize increasing wireless network demands, optimizing spectrum sensing, clustering algorithms, and energy-harvesting technology for cognitive-radio-based IoT networks in terms of deep-learning-based nonorthogonal multiple-access techniques. The nonorthogonal multiple-access scheme has high spectral efficiency and operates on the concept of signal superposition coding at the transmitter and sequential interference discontinuation at the receiver. Spectrum requests as a result of the mounting intensification in mobile data traffic are straightened out through exemplary multiple-access schemes for cognitive radio networks. Cognitive radio can diminish the consequences of spectrum under exploitation and scarceness resulting from the vast progression of wireless applications [41], optimizing both spectral and energy governance adequately. The adoption of internet of things devices, typically in huge volumes, across cutting-edge intelligent systems [105] has resulted in a massive wireless bandwidth and cognitive radio demand. Spectrum sensing can ensure a copresence between licensed users and unlicensed IoT devices [44] with the aim of streamlined spectrum utilization across cognitive-radio-based IoT communications.

## 8. Specific Contributions to the Literature

Our systematic review clarifies that cognitive radio is pivotal in the utilization of an adequate radio spectrum source [32], with spectrum sensing optimizing cognitive radio network operations; that opportunistic spectrum access and sensing [34] can boost the efficiency of cognitive radio networks; and that cooperative spectrum sharing together with simultaneous wireless information and power transfer [106] can increase spectrum and energy efficiency in 6G wireless communication networks and across IoT devices [107,108,109,110] for efficient data exchange. In addition, the 5G-enabled IoT articulates fast and low-latency data sharing and elevated communication coverage [111], attaining streamlined and sound massive node connections. Communication operations and persistence in cognitive radio networks [32] are significantly determined by accurate spectrum sensing function performance. Spectrum sensing is pivotal in cognitive radio technology due to the shadowing, fading, disturbance, and time-varying characteristics of wireless channels. Energy and matched filter detections are the most extensively deployed spectrum sensing strategies. The energy detector performance involves precise threshold expression selection in cognitive radio systems, where spectrum sensing performance is typified by leveraging the receiver operating characteristic curve.

Cognitive radio and nonorthogonal multiple access [18] are pivotal in 5G wireless networks. Harnessing nonorthogonal multiple-access techniques into cognitive radio networks can optimize spectrum efficiency and system capacity. Cognitive radio networks can assist in spectrum scarcity through spectral resource distribution between heterogeneous systems and users. Spectrum sharing across dense cognitive radio multicast networks is elaborate as a result of the strict interference limitations demanded by the primary users [10]: cognitive-radio-enabled multicasting ensures variability to service providers for enabling multicast group large-scale deployment in a spectrum-coherent fashion. As spectrum resource shortage limits 5G-enabled IoT advancement, cognitive radio together and nonorthogonal multiple access are suitable spectrum sharing technologies in spectrum use enhancement. Nonorthogonal multiple access configures increased deep-neural-network-based throughput and spectral efficiency [112] for 5G systems. A heterogeneous radio frequency configuration for wireless industrial networks [13] can enable various quality-of-service requirements of industrial IoT devices. A wireless sensor network across a sizable industrial facility integrates self-contained sensor devices deployed for analysis [15]: integrated computing, sensing, reliable communication, and distributed data processing technologies are pivotal in configuring sensor record supervision and transfer over a wireless network, correlating data perception, gathering, and handling in relation to perceived objects across the network coverage region. 

## 9. Limitations and Further Directions of Research

As we performed research based only on ProQuest, Scopus, and Web of Science sources and in the past few years, when we identified the most robust correlations, we possibly ignored some valuable research on optimizing spectrum sensing, clustering algorithms, and energy-harvesting technology for cognitive-radio-based IoT networks in terms of deep-learning-based nonorthogonal multiple-access techniques. Distinct review software systems and bibliometric network construction and visualization tools would also generate different outcomes. Future research should investigate the use of extended reality technologies and artificial-intelligence-based spectrum sensing, clustering, and energy harvesting in digital-twin-based virtual factories supported by cognitive-radio-based IoT networks.

## 10. Practical Implications

Long-range wide-area networks can assist IoT applications [113] by integrating large volumes of actively connected devices in process and environment monitoring and in process controlling. The received signal strength indicator-based localization can provide IoT device area data. Due to high bandwidth demands, cognitive radio technology [114] can assist unutilized communication spectrum bands for 5G wireless networks. In addition, the 6G-enabled IoT requires adequate spectrum resources [115] to offer large-scale IoT terminal spectrum access. Traditional orthogonal multiple access confines the entire utilization of limited spectrum resources: a nonorthogonal multiple-access-based hybrid spectrum access scheme can assist 6G-enabled cognitive IoT that can access both the inactive and active spectrum, notwithstanding the primary user state in relation to spectrum sensing, clustering algorithms, and energy-harvesting technology for cognitive-radio-based IoT networks in terms of deep-learning-based nonorthogonal multiple-access techniques. 

## Figures and Tables

**Table 1:** Topics and types of scientific products identified and selected.

Topic	Identified	Selected
cognitive-radio-based Internet of Things networks + spectrum sensing	119	26
cognitive-radio-based Internet of Things networks + clustering algorithms	116	25
cognitive-radio-based Internet of Things networks + energy-harvesting technology	112	24
Type of paper		
Original research	297	71
Review	16	4
Conference proceedings	26	0
Book	2	0
Editorial	6	0

Source: processed by the authors. Some topics overlap.

## Data Availability

Not applicable.
